# Chlorine-induced severe ARDS in an adolescent rescued with VV-ECMO: a case report with 6-month functional follow-up

**DOI:** 10.3389/fped.2026.1806880

**Published:** 2026-05-19

**Authors:** Huan Meng, Xiao-Fei Bi, Lei Wang, Xin-Tong Wang, Chun-Lai He, Hui-Ying Liu, Yu-Jia Tang, Xue Du, Wen Wei, Kai Kang, Li-Gang Si, Yang Gao

**Affiliations:** 1 Department of Critical Care Medicine, The Sixth Affiliated Hospital of Harbin Medical University, Harbin, Heilongjiang, China; 2 Department of Critical Care Medicine, The First Affiliated Hospital of Harbin Medical University, Harbin, Heilongjiang, China; 3 Department of Critical Care Medicine, The Traditional Chinese Medicine Hospital of Qiqihar City, Qiqihar, Heilongjiang, China; 4 Department of Pediatrics, The Sixth Affiliated Hospital of Harbin Medical University, Harbin, Heilongjiang, China

**Keywords:** acute respiratory distress syndrome, adolescent, chlorine intoxication, dual-lumen veno-venous extracorporeal membrane oxygenation, prophylactic antibiotics, pulmonary function

## Abstract

**Background:**

Chlorine is a potent irritant gas with asphyxiant toxicity. Depending on the concentration and duration of exposure, intentional or accidental inhalation can rapidly cause marked injury to the respiratory tract and, in some cases, leave patients with sustained ventilatory impairment. At present, there is no approved antidote that specifically targets chlorine intoxication. In this context, extracorporeal membrane oxygenation (ECMO) has been applied as a salvage therapy to provide temporary cardiopulmonary support for life-threatening respiratory failure that persists despite maximal conventional management. Among available configurations, veno-venous ECMO (VV-ECMO) delivered through a dual-lumen cannula (DLC) was designed to permit single-site cannulation and may lessen cannulation-related complications, reduce recirculation, and support earlier mobilization—including getting patients out of bed.

**Case presentation:**

To our knowledge, this is among the first reports in adolescents with longitudinal functional follow-up using chest computed tomography (CT) scans and pulmonary function tests in an adolescent with chlorine intoxication–induced severe acute respiratory distress syndrome (ARDS) who was successfully rescued with dual-lumen cannula veno-venous ECMO (DLC-VV-ECMO).

**Conclusion:**

In severe ARDS resulting from chlorine intoxication, VV-ECMO may function as a salvage strategy when conventional treatment is insufficient, providing temporary extracorporeal oxygenation and thereby preserving a window for pulmonary recovery and stabilization of other organ systems. During the acute phase of chlorine intoxication, prophylactic antibiotics are generally not recommended because the underlying lung injury is primarily caused by chemical insult and oxidative damage rather than established infection. Our findings also suggest that recovery of pulmonary function in adolescents after chlorine intoxication-induced severe ARDS may take longer than 6 months. Moreover, early suicide risk identification and timely mental health intervention are essential to prevent subsequent catastrophic outcomes.

## Introduction

Chlorine is a highly irritating, asphyxiant gas. After intentional or accidental exposure, inhalation can rapidly trigger a wide range of respiratory injuries—from upper-airway irritation and bronchial obstruction to increased vascular permeability, pulmonary edema, and, in severe cases, acute lung injury (ALI) and acute respiratory distress syndrome (ARDS). Clinical severity and the likelihood of persistent ventilatory impairment vary with both the concentration and the duration of exposure, and individuals with underlying chronic respiratory disease (including asthma) appear particularly vulnerable (1–4). A major component of this toxicity is oxidative injury: chlorine hydrolyzes on contact with moist airway surfaces, generating hypochlorous and hydrochloric acids that damage the respiratory epithelium and may set the stage for longer-term ventilatory dysfunction (5). Injury may also be amplified by chlorination by-products (CBPs), which form when chlorine reacts with natural organic matter and have been implicated in related toxic pathways (6). In routine practice, *β*2-agonists and corticosteroids are often administered for chlorine-induced respiratory injury, yet the evidentiary base remains modest. No specific antidote for chlorine intoxication has been approved by the U.S. Food and Drug Administration (FDA) to date (7, 8). Over recent years, chlorine exposure has drawn increasing clinical attention, in part because reports of chlorine-related incidents have risen in a sustained manner (9, 10). Even so, the full spectrum of life-threatening presentations and longer-term sequelae after chlorine intoxication is still incompletely described, with the knowledge gap especially apparent in children and adolescents (11, 12).

Extracorporeal membrane oxygenation (ECMO) is widely regarded as a salvage therapy, offering temporary cardiopulmonary support for patients with life-threatening respiratory failure and/or cardiogenic shock that does not respond to conventional management. As devices, expertise, and clinical workflows have matured, ECMO deployment has expanded rapidly across the globe and is now commonly used as a bridge to recovery or transplantation (13–16). One refinement is veno-venous ECMO (VV-ECMO) delivered via a dual-lumen cannula (DLC), which was introduced to simplify cannulation. Performed in the intensive care unit (ICU) under bedside imaging guidance—such as ultrasonography and/or portable radiography—this approach can avoid multiple access sites, reduce cannulation-related complications, limit recirculation, and support earlier mobilization, including out-of-bed activity (17–21). At the same time, ECMO remains a double-edged intervention. Contact between blood and the extracorporeal circuit may provoke immune activation and a systemic inflammatory response syndrome (SIRS), which can amplify inflammation and potentially worsen outcomes, particularly when layered onto the patient's underlying disease process (22). Sivelestat sodium (SIV), a selective neutrophil elastase (NE) inhibitor, has been proposed as a means to attenuate organ injury by suppressing NE activity and dampening downstream inflammatory cascades (23–25).

To the best of our knowledge, we report the first adolescent case of severe ARDS following chlorine intoxication that was successfully supported with VV-ECMO using a dual-lumen cannula. We also provide a staged six-month follow-up, tracking longer-term evolution on chest imaging alongside pulmonary function testing. This report underscores not only the acute organ injury that chlorine exposure can precipitate, but also the persistent respiratory consequences that may extend well beyond the initial crisis, and it offers clinical pointers for tailoring supportive care to lessen early injury, curb downstream morbidity, reduce mortality risk, and improve post-recovery quality of life.

## Case presentation

### Chief complaints

On April 23, 2025, a 15-year-old boy was transferred to the Department of Critical Care Medicine at the Sixth Affiliated Hospital of Harbin Medical University, a tertiary women's and children's hospital, for further recovery and rehabilitation. He had been successfully rescued from chlorine-induced severe ARDS with dual-lumen cannula veno-venous ECMO (DLC-VV-ECMO) in the Department of Critical Care Medicine at the First Affiliated Hospital of Harbin Medical University.

### History of present illness

**Figure 1:**
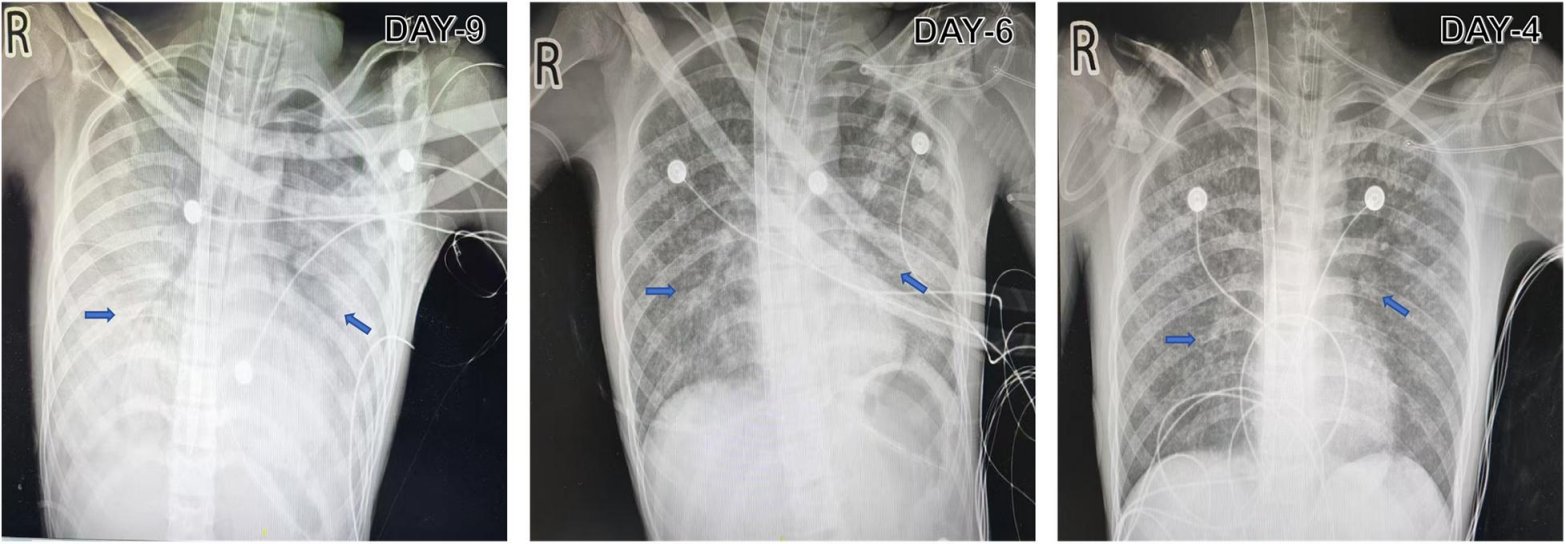
Chest anterior-posterior X-rays during hospitalization in the Department of Critical Care Medicine at the First Affiliated Hospital of Harbin Medical University. Serial chest radiographs obtained on days 9, 6, and 4 showed progressive improvement in bilateral pulmonary abnormalities. On day 9, diffuse bilateral high-attenuation pulmonary opacities produced a near-complete white-out appearance with visible air bronchograms. By day 6, prominent pulmonary markings and patchy ill-defined opacities were present in both lung fields. By day 4, the lungs showed prominent pulmonary markings with scattered bilateral pulmonary nodules, predominantly involving the upper lung fields.

**Figure 2:**
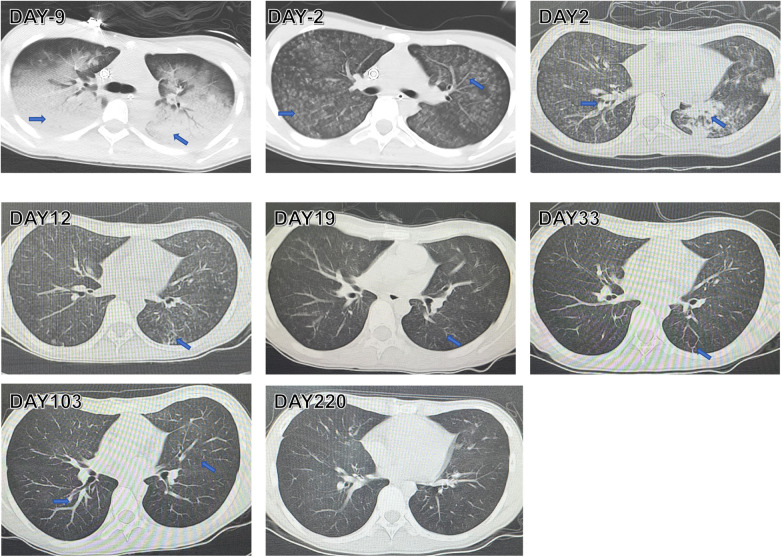
Chest CT scans before transfer, during hospitalization at the Sixth Affiliated Hospital of Harbin Medical University, and during staged follow-up. CT, computed tomography. Serial chest CT images obtained on days −9, −2, 2, 33, 103, and 220. Day −9 showed diffuse bilateral pulmonary opacification with a near-complete white-out appearance and visible air bronchograms. Day −2 showed preserved pulmonary architecture with bilateral high-attenuation opacities. Day 2 showed diffuse bilateral tree-in-bud opacities and peribronchial nodules, along with scattered small patchy and linear opacities and patchy consolidation in the left lower lobe. Day 33 showed persistent bilateral patchy and linear high-attenuation opacities. Day 103 showed residual left-predominant patchy and linear opacities, focal left pleural thickening, and a few tiny right lung nodules. By day 220, the small right lung nodules had resolved completely.

Ten days before admission, the adolescent was exposed to a large amount of chlorine gas that he had generated himself and was subsequently admitted to the Department of Critical Care Medicine at the First Affiliated Hospital of Harbin Medical University. He was diagnosed with severe ARDS caused by chlorine intoxication. Continuous analgesia and sedation were administered with remifentanil and midazolam. Invasive mechanical ventilation (IMV) was initiated using a lung-protective approach, and intermittent prone positioning was continued for 8 days. Despite mechanical ventilation with an inspired oxygen fraction of 100%, peripheral oxygen saturation remained between 59% and 77% on that day. The PaO2/FiO2 ratio (P/F) was 38.7 mmHg and remained at that level for 4 h, leading to initiation of VV-ECMO, which was maintained for 7 days. The system included a single-use dual-lumen venous cannula for a cardiopulmonary bypass system (MAQUET Cardiopulmonary, model 70126), an extracorporeal circulation tubing pack (MAQUET Cardiopulmonary, model BE-PLS2050), and extracorporeal circulation cannulae and puncture accessories (MAQUET Cardiopulmonary, model PIK150). Four days before admission, the gas inlet and outlet of the ECMO oxygenator were completely closed. The patient remained hemodynamically stable, and arterial blood gas results were satisfactory, after which decannulation was performed. High-flow nasal cannula (HFNC) oxygen therapy was then continued for 2 days. Antimicrobials comprised empirical biapenem for 7 days, followed by cefoperazone/sulbactam plus tigecycline for 3 days to treat *Acinetobacter baumannii* (AB). Intravenous methylprednisolone sodium succinate was given in a tapering regimen of 400 mg/day, 80 mg/day, and 40 mg/day, each for 3 days. SIV was administered at 4.8 mg/kg/day for 5 days to reduce the inflammatory response. Additional supportive care included transfusion of blood products for coagulopathy and vasopressor therapy. After successful discontinuation of DLC-VV-ECMO and IMV, followed by transition to HFNC, he was transferred to the Sixth Affiliated Hospital of Harbin Medical University for further recovery and rehabilitation. Laboratory findings and chest imaging obtained during his stay at the First Affiliated Hospital are presented in Table 1 and Figures 1, 2, respectively.

**Table 1:** Timeline of disease progression (April 13th to November 30th, 2025).

	The First Affiliated Hospital of Harbin Medical University/ICU										Hospitalization/ICU					Rehabilitation ward			After discharge		
day of illness	DAY-10	DAY-9	DAY-8	DAY-7	DAY-6	DAY-5	DAY-4	DAY-3	DAY-2	DAY-1	DAY0	DAY1	DAY2	DAY4	DAY6	DAY7	DAY12	DAY19	DAY33	DAY103	DAY220
Disease course																					
WBC (×10^9^/L)	43.26	22.69	27.15	21.83	10.08	10.94	10.83	10.74	16.26	9.54	30.21	23.09	5.96	11.12	15.97		7.35				
NEUT%	79.6	87.5	95	91	88.3	82.2	82.6	83.6	77.4	69.6	90.3	78.2	42	57.9	71.7		36.1				
NEUT (×10^9^/L)	34.42	19.87	25.77	19.88	9.43	8.99	8.94	8.98	12.58	6.63	27.28	18.04	2.5	6.44	11.45		2.65				
LYMPH% (%)	17.6	4.8	3.6	4.4	6.1	9.5	10.7	10.5	12.4	13.6	3.4	8.6	24.6	2.09	10.9		32.8				
HGB (g/L)	218	152	146	127	108	111	103	99	113	111	132	113	108	113	118		108				
HCT (%)	70.2	44.5	45.1	38.2	33.1	35.4	32	30.7	34.3	33.2	39.6	34.8	31.9	34.1	35.7		33.8				
PLT (×10^9^/L)	257	139	125	95	52	60	99	129	185	300	498	464	443	507	558		348				
PT (s)	25.7	15.8	16	14.1		13.7	11.7	11.7	11.6		15.1			11.5							
PT (%)	27.9	52.1	51.2	59.2		61.1	84.8	84.8	89		54.9			90.7							
INR	2.27	1.35	1.36	1.2		1.15	0.96	0.98	0.96		1.32			1							
APTT (s)	57.6	0	31.2	0		0	0	0	0		29.9			28.9							
FIB (g/L)	0.5	4.62	4.88	3.09		0.45	1.17	1.78	1.98		6.48			4.58							
TT (s)	36.1	18.2	15.6	17.6		28.3	21.3	18.9	18.6		14.5			14.9							
DD (mg/L)	72.12	26.79	69.73		40.91	41.68	34.85	29.03	12.16		2.09										
LDH(U/L)											450.09		299.32	198.27			254.12				
AST (U/L)	54.3	52.7	125.5		57.1	42.5		28.3			21		20	24	34		19				
ALT (U/L)	15.9	27.1	65.3		50.6	52.1		57.9			40.6		33.7	64.3	87.7		31.8				
ALB (g/L)	26.9	33.2	31.2		33.9	39.6		41.8			41.08		32.47	33.55	34.25		32.93				
BUN (mmol/L)	5.59	7.7	6.62								13.91		7.63				5.12				
SCr (*μ*mol/L)	84.8	85	60.6					41.1			45		35	40			41				
hs-cTnI (ng/L)	262.7		405.4		87.3				14.2		11										
CRP (mg/L)		14.3									113.59	111.78	40.99	15.89	4.66		0.91				
PCT (ng/mL)	4.77	24.38	16	9.14	5.61	3.06	1	0.32			2.97		2.12	0.63	0.38		0.06				
Oxygen therapy	Invasive mechanical ventilation							High-flow nasal cannula					Nasal catheter oxygen inhalation								
PEEP (cmH₂O)	6	5	8	8	8	8	8	5	5												
FiO₂	100	40	40	40	40	35	40	35	35	37	40	35	33	33	33	25					
pH	7.089	7.416	7.407	7.427	7.412	7.419	7.51	7.463	7.425	7.546	7.459	7.461	7.493	7.512	7.472	7.421					
PCO₂(mmHg)	55.2	42.2	46.5	45.2	49.1	47.8	38.1	37.8	42.9	31.9	37.4	38.2	41.2	44.1	38.6	40.6					
PO₂ (mmHg)	38.7	94.3	118	83.1	88.5	101	155	146	148	131	80.8	92.2	76.6	77.4	71.5	75.6					
P/F (mmHg)	38.7	235.75	295	207.75	221.25	288.57	387.5	417.14	422.86	354.04	202.2	263.43	232.12	234.55	216.67	302.4					
LAC (mmol/L)	7.1	2.4	1.4	1.7	2.1	2.5	1.8	1.2	1.1	1.3	0.9	1.1	1.5	2.3	1.9	0.6					
HCO_3_^−^(mmol/L)	13.6	26.3	27.7	28.6	29.5	29.5	30.8	27.2	27.4	29.2	26.7	27.3	31.3	34.7	28.3	25.9					
	Veno-venous extracorporeal membrane oxygenation						weaning of the ECMO														
Pump speed (r/min)	3,175	3,100	3,010	3,059	3,060	2,575	2,575														
Blood flow (L/min)	3.21	2.43	2.56	2.38	2.24	2.16	2.16														
Sweep gas flow(L/min)	3	3	3	3	3	2	0														
RALE score	28/48				16/48		12/48														
CT severity score		20/25							12/25				8/25				5/25	2/25	1/25	1/25	0/25
Antibiotics	biapenem							cefoperazone/sulbactam +tigecycline			cefoperazone/sulbactam										

WBC, white blood cell count; NEUT%, neutrophil proportion; NEUT, neutrophil count; LYMPH%, lymphocyte percentage; HGB, hemoglobin; HCT, hematocrit; PLT, platelet; PT(s), prothrombin time; PT(%), prothrombin activity; INR, international normalized ratio; APTT, activated partial thromboplastin time; FIB, fibrinogen; TT, thrombin time; DD, D-dimer; LDH, lactic dehydrogenase; AST, aspartate aminotransferase; ALT, alanine aminotransferase; ALB, albumin; BUN, blood urea nitrogen; SCr, serum creatinine; CRP, C-reactive protein; PCT, procalcitonin; PEEP, positive end-expiratory pressure; FiO2, fraction of inspired oxygen; PCO₂, partial pressure of carbon dioxide; PO₂, partial pressure of oxygen; P/F, PO₂/FiO₂ ratio; LAC, lactate; RALE score, radiographic assessment of lung edema score.

### History of past illness

On June 13, 2023, the adolescent was diagnosed with a pineal region germinoma complicated by obstructive hydrocephalus in the Department of Neurosurgery at the First Affiliated Hospital of Harbin Medical University, and a ventriculoperitoneal shunt was placed. From July 12 to November 22, 2023, he completed five cycles of chemotherapy in the Department of Oncology (carboplatin 700 mg on day 1 and etoposide 80 mg on days 2–4). He then underwent radiotherapy between November 22 and December 5, 2023, receiving 12 fractions of 2 Gy to the whole ventricle as the planning target volume (PTV) and 10 fractions of 2 Gy to the lesion as the clinical target volume (CTV), without spinal irradiation. On October 11, 2024, follow-up brain MRI demonstrated complete resolution of the initial lesion, and the tumor was considered in remission. No post-treatment pulmonary evaluation (e.g., chest imaging or pulmonary function tests) was performed after oncologic therapy. The patient reported no apparent limitation in daily activity and remained independent in activities of daily living. Over this extended treatment course, however, he gradually developed persistent low mood and hopelessness; these changes were not sufficiently recognized by his legal guardians, and he did not access professional mental health care.

### Personal and family history

There was no relevant personal or family history.

### Physical examination

Upon admission, he was 1.70 m tall and weighed 40 kg [body mass index (BMI), 13.84 kg/m^2^]. His vital signs were: temperature 36.5 °C, heart rate 134 beats/min, blood pressure 90/40 mmHg, respiratory rate 48 breaths/min, and pulse oxygen saturation 94%. On examination, he appeared listless and markedly tachypneic, with rapid, shallow breathing. Petechiae were present on the left bulbar conjunctiva. Breath sounds were reduced over the left lung, and scattered dry and wet rales were heard bilaterally. Extensive ecchymoses were noted in both groin regions. Multiple healed surgical scars were visible on the scalp, and a ventriculoperitoneal shunt was palpable beneath the right temporal scalp.

### Laboratory parameters

Laboratory parameters obtained prior to transfer and during hospitalization at the Sixth Affiliated Hospital of Harbin Medical University are presented in Table 1. Arterial blood gas analysis showed a pH of 7.466, an ionized calcium level of 1.10 mmol/L, and a glucose level of 7.6 mmol/L, without other notable abnormalities. Qualitative nucleic-acid testing was negative for human rhinovirus, adenovirus, Mycoplasma pneumoniae, respiratory syncytial virus, and influenza A/B. The Pediatric Critical Illness Score (PCIS) on admission was 92.

### Imaging findings

Upon admission, echocardiography and abdominal ultrasonography were unremarkable apart from mild hepatomegaly, with the liver edge palpable 1.7 cm below the right costal margin. Chest anterior-posterior X-rays, chest computed tomography (CT) scans obtained before transfer, during hospitalization at the Sixth Affiliated Hospital of Harbin Medical University, and at prespecified follow-up visits are presented in Figures 1, 2.

### Diagnosis upon admission

Based on a comprehensive assessment of the present illness and past medical history, clinical manifestations, physical examination, laboratory findings, and imaging upon admission, the adolescent was diagnosed with secondary pneumonia after chlorine intoxication-induced severe ARDS, with concomitant coagulopathy and elevated myocardial enzyme levels.

### Treatment and follow-up

Upon admission to the Department of Critical Care Medicine at the Sixth Affiliated Hospital of Harbin Medical University, treatment was initiated without delay. Continuous analgesia with light sedation was provided using remifentanil and dexmedetomidine, and HFNC was maintained. The patient was intermittently repositioned to the left and right lateral decubitus positions. Empiric cefoperazone/sulbactam was started for suspected AB infection. Methylprednisolone sodium succinate was administered intravenously (40 mg once daily for 3 days, followed by 30 mg once daily for 4 days). Prophylactic low-molecular-weight heparin (4,000 IU subcutaneously once daily) was given for deep vein thrombosis (DVT) prevention. Enteral nutrition via a nasogastric tube (1.5 kcal/mL, 1,000 mL/day) was provided, along with potassium citrate (three packets/day). A conservative, de-escalation fluid strategy was adopted. Ambroxol was used as a mucolytic, and nebulized terbutaline sulfate combined with budesonide was administered. Induced sputum was collected for targeted next-generation sequencing (tNGS) and routine culture.

On hospital day 2, repeat chest CT demonstrated diffuse tree-in-bud opacities and peribronchial nodules in both lungs, along with scattered small patchy and linear opacities and patchy consolidation in the left lower lobe (Figure 2). Targeted next-generation sequencing (tNGS) of induced sputum identified AB, *Klebsiella pneumoniae* (KP), and *Pseudomonas aeruginosa* (PA); the normalized read counts (×10^6) were 35,876, 34,963, and 18,078, respectively. The resistance gene *bla*CTX-M was detected and was considered likely attributable to KP and PA. With clinical improvement, HFNC support was gradually weaned on day 2 and the patient was transitioned to conventional nasal cannula oxygen. In parallel, induced-sputum culture yielded ESBL-producing KP and carbapenem-resistant AB (CRAB), and both isolates remained susceptible to cefoperazone/sulbactam. On hospital day 4, the rehabilitation team initiated early rehabilitation, including respiratory training, exercise therapy, and microneedle acupuncture. On hospital day 5, the first pulmonary function test showed severe combined obstructive and restrictive ventilatory impairment (FEV1/FVC 75.70% (z = −2.0), FEV1 0.93 L (26% predicted, z = −8.70), FVC 1.23 L (30% predicted, z = −10.0), VCmax 1.21 L (29% predicted)) (Table 2).

**Table 2:** Pulmonary function tests during hospitalization at the Sixth Affiliated Hospital of Harbin Medical University and phased follow-up.

day of illness	DAY5				DAY33				DAY103				DAY221			
Pulmonary function during forced expiration	Predicted value	Measured value	Measured/Predicted (%)	z-scores	Predicted value	Measured value	Measured/Predicted (%)	z-scores	Predicted value	Measured value	Measured/Predicted (%)	z-scores	Predicted value	Measured value	Measured/Predicted (%)	z-scores
FVC (L)	4.18	1.23	30	−10	4.18	1.81	43	−8	4.3	2.33	54	−6.4	4.3	2.99	69	−4.2
FEV1 (L)	3.61	0.93	26	−8.7	3.61	1.2	33	−7.9	3.71	1.62	44	−6.7	3.71	1.98	53	−5.6
FEV1/FVC (L)	86.85	75.7	87	−2	86.85	66.22	76	−3.4	86.69	69.43	80	−2.9	86.69	66.28	76	−3.4
FEF25 (L/s)	6.14	1.9	31	−2.1	6.14	1.7	28	−2.2	6.3	2.59	41	−1.8	6.3	2.73	43	−1.7
FEF50 (L/s)	4.36	0.89	20	−2.5	4.36	1.02	23	−2.4	4.47	1.26	28	−2.2	4.47	1.6	36	−2.0
FEF75 (L/s)	2.36	0.4	17	−5.2	2.36	0.39	17	−5.3	2.42	0.53	22	−4.6	2.42	0.59	24	−4.4
FEF25-75 (L/s)	4.05	0.68	17	−6.1	4.05	0.78	19	−5.8	4.13	1.16	28	−4.8	4.13	1.38	33	−4.3
PEF (L/s)	7.11	2	28	−2.1	7.11	2.6	37	−1.9	7.31	3.07	42	−1.7	7.31	2.85	39	−1.8
Slow Vital Capacity																
VC MAX (L)	4.2	1.21	29		4.2	1.69	40		4.34	2.69	62		4.34	3.04	70	
VT (L)	0.58	0.41	70		0.58	0.56	96		0.59	0.74	124		0.59	0.66	112	
ERV (L)	1.45	0.54	37		1.45	0.66	45		1.5	1.19	80		1.5	1.25	83	
IRV (L)	2.18	0.26	12		2.18	0.47	22		2.25	0.76	34		2.25	1.12	50	
IC (L)	2.71	0.67	25		2.71	1.03	38		2.79	1.5	54		2.79	1.79	64	
VC IN (L)	4.2	0.68	16		4.2	1.04	25		4.34	1.67	38		4.34	1.78	41	
VC EX (L)	4.2	1.21	29		4.2	1.69	40		4.34	2.69	62		4.34	3.04	70	
MVV																
VT (L)	0.58	0.36	61		0.58	0.35	61		0.59	0.48	82		0.59	0.44	74	
BF (1/min)	20	105	527		20	110	548		20	112	561		20	115	577	
MVV (L/min)	72.46	37.52	52		72.46	38.63	53		75.08	54.31	72		75.08	50.24	67	

FVC, forced vital capacity; FEV1, forced expiratory volume in the first second; FEV1/FVC, one-second rate; FEF25, forced expiratory flow at 25% of vital capacity; FEF50, forced expiratory flow at 50% of vital capacity; FEF75, forced expiratory flow at 75% of vital capacity; FEF25-75, mid-expiratory flow rate; PEF, peak expiratory flow; VC MAX, maximal vital capacity; VT, tidal volume; ERV, expiratory reserve volume; IRV, inspiratory reserve volume; IC, inspiratory capacity; VC IN, inspiratory vital capacity; VC EX, expiratory vital capacity; MVV, maximum voluntary ventilation; BF, breathing frequency.

On hospital day 7, the adolescent's vital signs were stable, and he was transferred to the Department of Rehabilitation for continued recovery. Management largely mirrored the ICU strategy, including intravenous cefoperazone/sulbactam and intravenous methylprednisolone sodium succinate (30 mg/day for 3 days, then 25 mg/day for 3 days), followed by a tapering course of oral methylprednisolone sodium succinate (20 mg/day, 12 mg/day, and 6 mg/day, each for 2 days). Rehabilitation measures consisted of respiratory training, exercise therapy, and microneedle acupuncture. Ambroxol was administered to facilitate sputum clearance, and terbutaline sulfate plus budesonide suspension was delivered via nebulization. On hospital day 10, an induced sputum culture grew carbapenem-resistant *Klebsiella pneumoniae* (CRKP), which was resistant to cefoperazone/sulbactam. Two days later (hospital day 12), induced sputum cultures again yielded carbapenem-resistant *Pseudomonas aeruginosa* (CRPA) and carbapenem-resistant *Acinetobacter baumannii* (CRAB), both of which remained susceptible to cefoperazone/sulbactam. A repeat chest CT scan demonstrated improvement of the patchy consolidation in the left lower lobe (Figure 2). Cefoperazone/sulbactam was discontinued on hospital day 14.

### Outcome and follow-up

The patient was discharged on day 19 of hospitalization and was advised to continue pulmonary rehabilitation after discharge, with repeat pulmonary function tests and chest CT scans scheduled at 2 weeks, 3 months, and 6 months post-discharge. At 14 days post-discharge, chest CT revealed bilateral high-attenuation opacities with patchy and linear distributions; ill-defined consolidation persisted in some regions, most prominent in the left lower lobe (Figure 2). Pulmonary function testing demonstrated severe obstructive ventilatory impairment with a concomitant moderate restrictive defect (FEV1/FVC 66.22% (z = −3.4), FEV1 1.2 L (33% predicted, z = −7.9), FVC 1.81 L (43% predicted, z = −8.0), VCmax 1.69 L (40% predicted)) (Table 2). By 84 days after discharge, CT showed residual patchy and linear opacities confined mainly to the left lung, focal thickening of the left pleura, and a few tiny nodules in the right lung. Pulmonary function results were broadly unchanged—still severe obstruction with moderate restriction—although several indices had inched upward (FEV1/FVC 69.43% (z = −2.9), FEV1 1.62 L (44% predicted, z = −6.7), FVC 2.33 L (54% predicted, z = −6.4), VCmax 2.69 L (62% predicted))(Table 2). At 201 days post-discharge, chest CT documented complete disappearance of the small right-lung nodules (Figure 2). Pulmonary function testing had improved to moderate obstructive and restrictive ventilatory dysfunction (FEV1/FVC 66.28% (z = −3.4), FEV1 1.98 L (53% predicted, z = −5.6), FVC 2.99 L (69% predicted, z = −4.2), VCmax 3.04 L (70% predicted)) (Table 2).

## Discussion

At the onset of the disease, severe ARDS secondary to chlorine intoxication was diagnosed on the basis of the patient's medical history, clinical manifestations, physical examination, laboratory findings, and imaging results. In the presence of persistent severe hypoxemia despite conventional mechanical ventilation, lung-protective ventilation, and comprehensive supportive care, VV-ECMO may serve as an important salvage therapy by temporarily replacing pulmonary gas exchange and thereby allowing time for lung recovery, inflammation control, and stabilization of organ function (26). Because lung injury in this setting is driven primarily by chemical insult and oxidative damage rather than true infection, prophylactic antibiotics are generally not recommended (27–29). Against this background, early prophylactic administration of biapenem, a carbapenem antibiotic, may have imposed selective pressure and contributed to the subsequent isolation of carbapenem-resistant organisms in induced sputum, including CRAB, CRKP, and CRPA. More broadly, initiation and modification of antimicrobial therapy should not rely solely on culture results, particularly when specimens are obtained from a nonsterile airway; instead, treatment decisions should be guided by the overall clinical course, laboratory trends, and radiographic changes (29). This reasoning explains why antimicrobial therapy was not escalated on hospital day 10 despite an induced-sputum culture yielding CRKP resistant to cefoperazone/sulbactam, and why detection of CRPA and CRAB in induced sputum on hospital day 12 likewise did not alter the decision to discontinue cefoperazone/sulbactam on hospital day 14. During the acute phase of chlorine intoxication, the child also developed myocardial injury, a finding consistent with previous studies showing that chlorine toxicity is not confined to the lungs but may also involve the myocardium, manifesting as myocardial depression, hemodynamic instability, and cardiac dysfunction, possibly mediated by oxidative stress and mitochondrial injury (8). Accordingly, in chlorine intoxication, monitoring for myocardial injury and cardiac dysfunction should be emphasized in addition to respiratory support.

Although the combined regimen—*β*2-agonist therapy, systemic corticosteroids, SIV, and VV-ECMO delivered via a dual-lumen cannula—successfully rescued this adolescent with chlorine intoxication–induced severe ARDS, the apparent benefit should be viewed cautiously and confirmed in well-designed studies. Follow-up with serial chest CT and pulmonary function tests showed a striking mismatch: radiographic abnormalities largely resolved, yet lung function remained moderately compromised, with mixed obstructive and restrictive ventilatory defects. In cases of chlorine intoxication, recovery appears to follow the pattern already described for other causes of ARDS, in which pulmonary function may remain impaired for months after hospital discharge. Additional work is needed to define the long-term pulmonary sequelae of chlorine intoxication in this age group and to clarify the mechanisms involved, so that recovery-oriented strategies can be better targeted and quality of life improved.

In this case, the diagnosis of pineal region germinoma with obstructive hydrocephalus, together with subsequent surgery, chemotherapy, and radiotherapy, may have contributed to the emergence of suicidality in this adolescent. However, this risk was not sufficiently recognized by the legal guardians. Because suicidality can progress from psychosocial stress and emotional dysregulation to suicidal ideation and suicidal behavior, early recognition and timely preventive intervention are crucial (30–32). Greater attention should therefore be directed toward the early identification of suicide-related risk factors, careful screening of at-risk individuals, promotion of help-seeking behaviors, reinforcement of social support, and timely referral for evidence-based, person-centered mental health care (33–35).

## Conclusion

Taken together, in severe ARDS resulting from chlorine intoxication, VV-ECMO may function as a salvage strategy when conventional treatment is insufficient, providing temporary extracorporeal oxygenation and thereby preserving a window for pulmonary recovery and stabilization of other organ systems. During the acute phase of chlorine intoxication, prophylactic antibiotics are generally not recommended because the underlying lung injury is primarily caused by chemical insult and oxidative damage rather than established infection. Our findings also suggest that recovery of pulmonary function in adolescents after chlorine intoxication-induced severe ARDS may take longer than 6 months. Moreover, early suicide risk identification and timely mental health intervention are essential to prevent subsequent catastrophic outcomes.

## Data Availability

The original contributions presented in the study are included in the article/supplementary material, further inquiries can be directed to the corresponding authors.
